# A High Power-Conversion-Efficiency Voltage Boost Converter with MPPT for Wireless Sensor Nodes

**DOI:** 10.3390/s21165447

**Published:** 2021-08-12

**Authors:** Xiwen Zhu, Qiang Fu, Ruimo Yang, Yufeng Zhang

**Affiliations:** 1 MEMS Center, Harbin Institute of Technology, Harbin 150001, China; 18147651234@163.com (X.Z.); qiangfu.hit@gmail.com (Q.F.); y932829159@163.com (R.Y.); 2 Key Laboratory of Micro-Systems and Micro-Structures Manufacturing (Harbin Institute of Technology), Ministry of Education, Harbin 150001, China

**Keywords:** WSNs, photovoltaic cells, voltage boost converter, charge pump, MPPT

## Abstract

A high power-conversion-efficiency voltage boost converter with MPPT for wireless sensor nodes (WSNs) is proposed in this paper. Since tiny wireless sensor nodes are all over complex environments, an efficient power management system (PMS) must be equipped to achieve long-term self-power supply and maintain regular operation. It is common to use Photovoltaic cells (PV) to harvest sunlight in the environment. However, most existing interface boost integrated circuits for the PV cell have low efficiency. This paper presents a voltage boost converter (VBC) with high power conversion efficiency (PCE) for WSNs. The integrated circuit (IC) designed in this paper includes a novel four-phase high-efficiency charge pump module, an ultra-low-power perturbation observation (P&O) MPPT control circuit module, a feedback control module, a nano-ampere current reference, etc. Manufactured in a standard 0.35 um complementary metal-oxide-semiconductor (CMOS) technology, the chip area is 3.15 mm × 2.43 mm. Test results demonstrate that when the output voltage of the PV cell is more than 0.5 V, VBC can improve the voltage to 3Vin, and the calculated voltage conversion efficiency can reach 99.4%. P&O MPPT algorithm makes output power improving 8.53%. Furthermore, when the output load current is 297uA, the output PCE achieves 85.1%.

## 1. Introduction

It is acknowledged that the wireless network node, as a microsensor, is generally composed of four parts, the energy supply part, sensor part, processor part, and wireless communication part. The energy supply part provides stable voltage to the other three ones. Many WSNs work in complex application scenes, such as traffic monitoring, intelligent households, etc. As a result, the replacement of batteries becomes challenging to implement. Wireless sensor network nodes tend to collect energy in the environment to ensure long-term stable operation. The most versatile energy source is solar power, having wide distribution, pollution-free, almost inexhaustible advantages. Therefore, photovoltaic power generation is one of the most rapidly developing technologies in recent years. However, the power generated by PV cells is directly proportional to the temperature and light intensity, which cannot be kept constant. Besides, the wireless sensor network nodes need to store the light energy collected by PV cells during the sunshine period, to realize all-weather self-power supply at the nodes, namely working at night [1].

Miniaturizing and having complete functions are the research goal of wireless sensor network nodes. Despite Energy harvesters have smaller and smaller sizes, most institutions do not design PMS circuits in an integrated method or with fundamental functions. Few institutions design the IC of voltage boost converter, whose Power-Conversion-Efficiency is low, not equipped with MPPT, and unsuitable for photovoltaic cells [2,3,4]. To solve these problems, more and more researchers engaged in this field. Michael D and his team from the University of California, Berkeley, designed an ultra-low-power power management chip for wireless sensor nodes in 2007. The power management chip can power the sensors and the radio. And the efficiencies are 75% and 65%, respectively. Although it cannot apply to photovoltaic battery systems, it provides valuable on-chip integration and a voltage multi-channel method [5]. Toshihiro Ozaki of Kobe University in Japan proposed a high PCE voltage boost converter with MPPT to collect low-voltage energy in 2015. In the design, the voltage boost by a voltage charge pump. The circuit can convert a minimum of 0.21 V input to a triple output, and the efficiency of the design reaches 73.6% [6]. Besides, the Kobe University team proposed a fully integrated VBC with MPPT in 2016 [7]. In 2015, Jungmoon Kim proposed a weak energy harvesting chip. In the design, they proposed a charge pump structure with adaptive dead zone and dynamic body bias. It can collect the low voltage of 0.15 V, and the maximum conversion efficiency has reached 72.5%. Nevertheless, it has not applied to photovoltaic battery systems, either [8]. Besides, some designs chase a high PCE by inductance, which takes up a large area [9,10,11,12,13].

This work presents a high Power-Conversion-Efficiency VBC for PV cells in WSNs. A novel four-phase high-efficiency charge pump structure is adopted to improve photovoltaic cells’ conversion efficiency and output power. We design the hill-climbing MPPT algorithm with low power to track the maximum power point and improves efficiency [14,15,16]. We install a nano-ampere current reference circuit to minimize power consumption. Moreover, a current-starved ring oscillator is used to improve the accuracy of the feedback control. We use 0.35 um CMOS process technology to make the device. Measured results are shown in part three, and the maximum output efficiency can achieve 85.1%.

This paper is organized as follows. Section 2 proposes modeling of Photovoltaic Cells and MPPT. The design of the VBC system is described in Section 3. Section 4 shows experimental results. Furthermore, in Section 5, the conclusions are illustrated.

## 2. System Design and Analysis

### 2.1. Modeling of the Photovoltaic Cell

**Figure 1:**
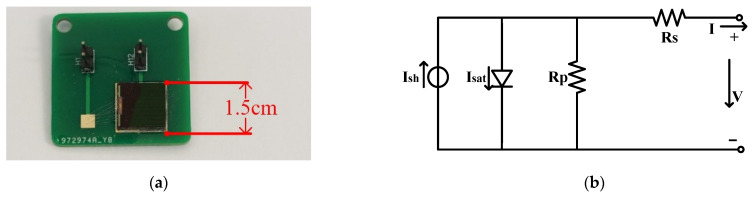
(**a**) Photograph of the PV cell; (**b**) Equivalent circuit diagram of a PV cell.

When sunlight irradiates a Solar photovoltaic cell, the photons are absorbed by the photovoltaic cell to generate electron-hole pairs due to the photovoltaic effect. As a result, the electrons and holes move to the depleting zone. And then, under the influence of the electric field in the depletion zone, the electrons and holes are moved to the negative and positive zone, respectively. Consequently, a potential difference is formed, and the PV cell converts solar energy into electrical energy [17]. Figure 1a shows the PV cell used in the platform, which is 1.5 cm × 1.5 cm and equivalent to the circuit as Figure 1b.

Combined with actual testing, we complete system-level modeling of photovoltaic cells. About a PV cell, the output current can be expressed as Equation (1).

(1) $$I=I_{ph}-I_{sat}\{\exp[\frac{q(V+IR_{s})}{AKT}-1]-\frac{V+IR_{s}}{R_{p}}\}-I_{sh}$$

where *I_ph_* is the sunlight-generated current, which is proportional to the light intensity; *I_sat_* is the reverse saturation current, which is related to temperature; *I_sh_* is the short-circuit current; *T* is the temperature of the photovoltaic cell; *q* is the charge of an electron; *A* is the ideal factor of the *PN* junction; *K* is the Boltzmann constant; *R_p_* is the equivalent parallel resistance; *R_s_* is equivalent series resistance. *I_ph_* varies with light intensity as Equation (2).

(2) $$I_{ph}=I_{sc}\times (\frac{S}{1000})+C_{T}\times (T-T_{ref})$$

where *S* is the light intensity; *I_sc_* is the short circuit current; *T_ref_* is the absolute temperature; *T* represents the battery temperature; *C_T_* is the temperature coefficient.

(3) $$I_{sh}=\frac{U_{out}+IR_{s}}{R_{sh}}$$

Equation (1) can be written as

(4) $$I=I_{sc}(\frac{S}{1000})+C_{T}(T-T_{ref})-I_{sat}\{\exp[\frac{q(V+IRs)}{AKT}-1]-\frac{V+IR_{s}}{R_{p}}\}-\frac{U_{out}+IR_{s}}{R_{sh}}$$

Ignore its parasitic parameters, and current can be obtained as

(5) $$I=I_{ph}-I_{sat}\exp[\frac{q(V+IRs)}{AKT}-1]\;$$

**Figure 2:**
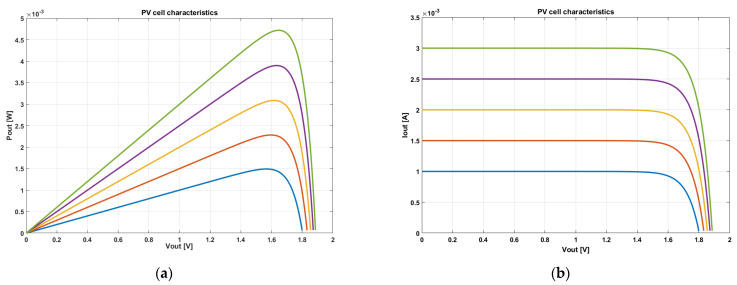
(**a**) Power/voltage curve of PV; (**b**) Current/voltage curve of PV.

After simulation of the system-level mathematical model, the output characteristics of photovoltaic cells under different illumination are shown in Figure 2. When the load current is large, and the voltage is small, a PV cell can be equivalent to a current source at this time. On the contrary, while the load impedance is large and the output voltage tends to be large and unchanged, it can be equivalent to a voltage source [18,19].

### 2.2. Analyze of MPPT Algorithm

Tracking the maximum power point (MPP) precisely is the key to improve power conversion efficiency. Adding an impedance converter to the output end of the PV cell is the fundamental principle of the MPPT algorithm. The impedance converter needs to measure the PV cell’s Certain output parameters, such as voltage, current, power, etc. Besides, it is necessary to consider the output impedance of the PV cell and the system’s power consumption to complete the maximum power point tracking. 

Standard MPPT algorithms discussed are as follows: Mathematical model optimization method, increment conductance method, perturbation observation method, and Intelligent processing and nonlinear control methods.

The self-optimization method we use in this paper is the perturbation observation method (P&O). Its basic principle is to give a perturbation to the output of the PV cell in each cycle, that is, to change the output impedance in each process. The output power change is judged, the direction of the next disturbance is given, and the maximum power point is finally found. P&O method has certain advantages, such as simple hardware implementation, fewer calculation parameters (current, voltage) to be detected, and real-time tracking of PV cells, with lower cost. It also has obvious shortcomings: in the process of disturbance observation, the disturbance will continue to work, causing the final operating point to oscillate slightly near the maximum power point. When the set disturbance voltage amplitude is small, the ripple characteristics of the output voltage will be significantly improved. The tracking accuracy is higher, but relatively, the time required to reach a stable state becomes longer. On the contrary, when the disturbance voltage amplitude is set larger, the response speed is faster, but the accuracy is significantly reduced, so it needs to choose an appropriate step size for the state. 

Note that the PV cell is the input component of the system. As a result, the output of the PV cell is the input of the VBC chip, and power consumed by the boost module cannot be ignored. The output power of the whole system can be described as

(6) $$P_{sys\_out}=P_{pv\_out}-P_{d}$$

where *P_d_* is the power loss during the boosting process, *P_sys_out_* is the system’s output power, and *P_pv_out_* is the output power of the photovoltaic cell.

**Figure 3:**
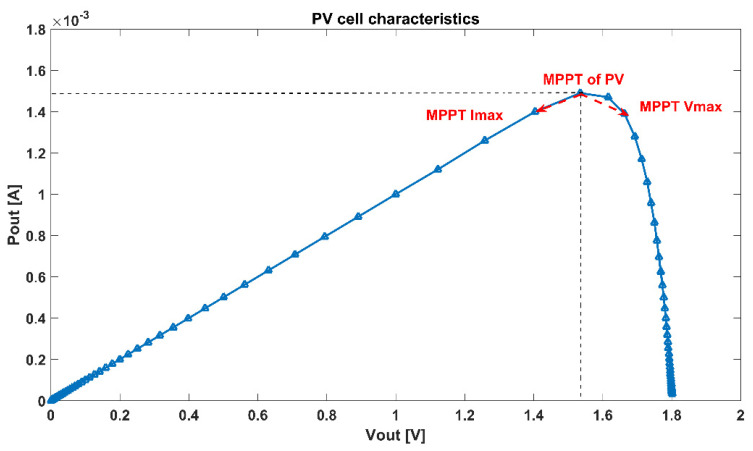
Schematic Curve Diagram of System MPPT.

When the output load of the system is constant, the maximum power means the maximum voltage. In another case, when the output load of the system is capacitive, the maximum current means the fastest charging speed. The MPP of the photovoltaic cell can provide the maximum input power, namely *P_pv_out_*. We design the charge pump modulated by PFM. Reducing the frequency *P_d_* can further reduce loss and *P_sys_out_* further increases, as shown in Figure 3. When Equation (7) is established, the positions of the two power points are different. As a result, we hope that when the output voltage reaches the maximum value in practical applications, the maximum power point will move to the right.

(7) $$\frac{dP_{d}}{df}=\frac{dP_{pv\_out}}{df}$$

**Figure 4:**
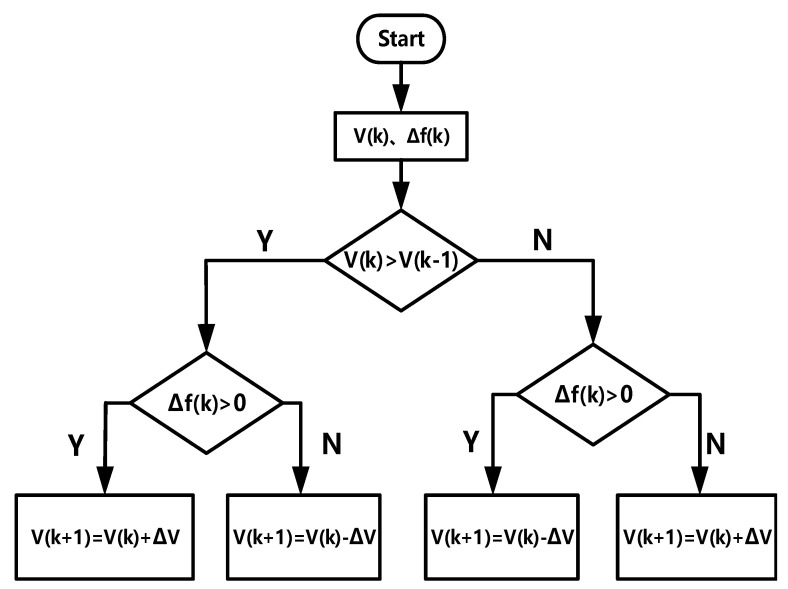
Logical block diagram of P&O MPPT.

Based on the above analysis, the logic block diagram of the disturbance observation method of this design is shown in Figure 4. It is an XOR relationship between the change of output voltage and the direction of voltage disturbance. 

In Figure 4, V(k) represents the system output voltage, ∆f(k) represents the adjusted clock frequency, and its essence is the same as ∆V(k). When the frequency change increases the system output voltage, MPPT will Keep this change. On the contrary, it will make this change in the opposite direction. In summary, MPPT has a more macro definition. Once the MPPT controller is put into the power management system for analysis, it can track the maximum power point, maximum voltage point, or maximum current point [20,21].

In conclusion, the disturbance observation method is used in this design to make the boost chip fully integrated while ensuring the efficiency of MPPT tracking, and it is as easy to implement as possible on the hardware. This design adopts the disturbance observation method, and the specific algorithm flow is introduced in detail in Section 3.

### 2.3. Design of the VBC System with MPPT

In the application of wireless sensor network nodes, it is necessary to realize the miniaturization of the structure and the full integration of the power management chip. Despite DC-DC converters having various advanced low-voltage start-up technologies, which can complete low-input voltage conversion and significantly improve conversion efficiency, the apparent disadvantage is large-area components such as inductors. On the contrary, although the transmission loss of the charge pump is larger than DC-DC converters, the boost converter in the charge pump method has more significant superiority in saving area.

**Figure 5:**
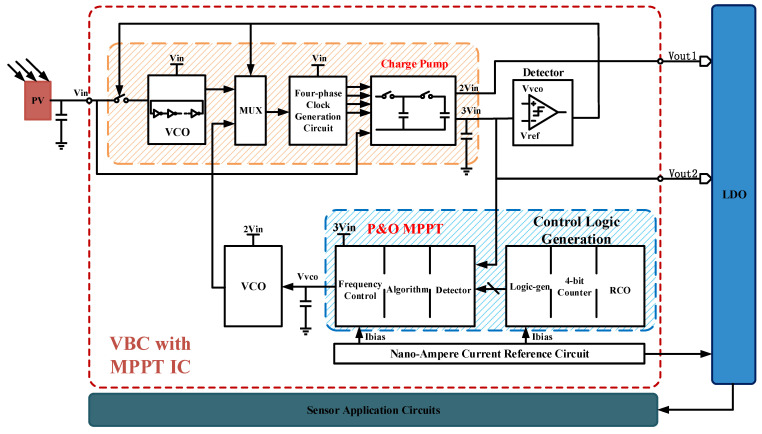
Block diagram of VBC with MPPT.

As a result, the design uses a charge pump as the main body of the boost converter. Figure 5 shows the overall boost converter logic diagram we proposed. The IC system we design includes a high PCE four-phase boost charge pump, a P&O MPPT control module, a nano-ampere current reference, a high-precision comparator, a four-phase clock generation circuit, and a current-starved VCO, etc. The whole IC is a closed-loop system, which can track maximum PowerPoint by self-detection. The *V_out_* of VBC will be given to LDO, and then the regulated voltage can be used in sensor application circuits. This power system management can apply in small WSNs powered by PV cells.

The proposed VBC with MPPT work can be divided into two stages. The first one is starting mode, the photovoltaic cell output voltage, same as system input voltage (0.5 V~1.8 V), gives energy to the ring oscillator (RCO), and provides power for the four-phase clock generation circuit and data selector; thus, a four-phase clock is generated. Then, the charge pump is driven to boost the input voltage and output 2 Vin and 3 Vin. While the output voltage is stable, the charge pump output continues to provide power for the MPPT module and the clock control module, and the *I_ref_* of the nano-ampere bias circuit module is generated and maintained stable. The detection circuit detects the reasonable value of the feedback voltage and controls the data selector to start the second stage and stop the ring oscillator. The detection circuit is an ultra-power comparator. 

The second stage is MPPT working mode. After the MPPT module works, it will make the voltage control oscillator (VCO) oscillates and generate a new clock. The clock frequency tracks its maximum value according to the change of the output voltage. The channel selector turns off the previous RCO, chooses the new clock to form a feedback loop with MPPT function, and finally stabilizes the charge pump output voltage at 3 Vin.

## 3. Circuit Block Implementation Details

### 3.1. High PCE Charge Pump

The charge pump is the most critical core module to the VBC. It uses the accumulation and release of charge in the capacitor to generate high voltage. The efficiency of the conventional Dickson charge pump is minimal due to body-effect, and diodes are difficult to manufacture in actual situations [22,23,24,25,26]. 

**Figure 6:**
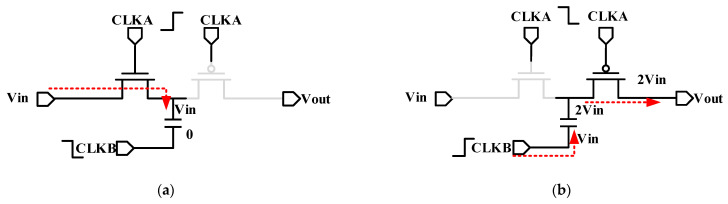

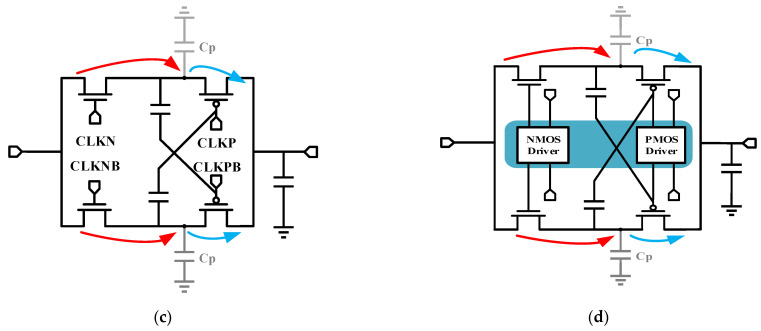
(**a**) A single charge pump voltage transfer schematic; (**b**) As single charge pump charging schematic; (**c**) Circuit diagram of Cross-coupled CMOS charge pump; (**d**) Circuit diagram of Cross-coupled CMOS charge pump with drivers.

Figure 6a,b illustrate the charging and discharging process of a simple charge pump. A pair of CMOS structures is used as a switch, which is controlled and driven by timing. CLKA and CLKB are two-phase non-overlapping clocks; the amplitude is VDD. In a single period, the capacitance is charged twice, and the upper plate is 2 Vin. We can derive that CP has doubled the voltage. Besides, the charge pump is cascaded into a multi-stage charge pump to obtain a high voltage boost multiple. However, it will result in a significant reduction in conversion efficiency. Since the cascade structure only makes the last stage output impedance in effect, and the output impedance is limited. Thus, we hope to increase the output impedance as much as possible.

As shown in Figure 6c, we can avoid a significant drop in the threshold voltage in low-voltage applications by using cross-coupled CMOS for boosting. In addition, the dual-channel structure of the upper and lower channels can also reduce the output ripples of the two channels, thereby improving the stability of the output voltage. Driving this type of charge pump needs a four-phase clock to avoid charge leakage. Figure 6d shows that we use CMOS drivers to improve the grid voltage of switches. The impedance of switches can be reduced, and the efficiency of CP rises further. 

**Figure 7:**
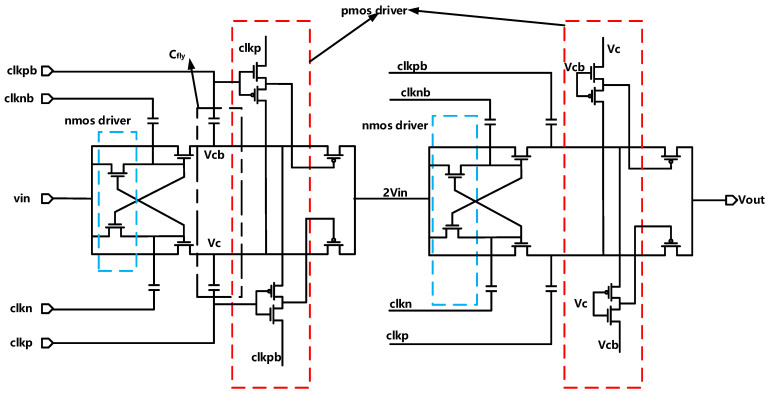
Schematic of the four-phase charge pump.

The charge stagnation time on the capacitor needs to be considered. The electric charge cannot be transferred instantaneously at the coupling capacitor under non-ideal conditions. Thus the charge on the capacitor will stagnate and cause consumption, and the output current waveform contains current spikes with finite pulse width. In addition, since the value of the on-resistance will be affected by the input signal, distortion related to the input signal will happen in the charge pump circuit. This paper uses a four-phase clock charge pump to solve the above problems effectively. It uses a two-stage dual-channel structure to achieve a voltage increase of three times. To reduce the voltage loss, the CMOS switch increases the drive to lower its on-resistance and the leakage current of the switch. The NMOS drives are CP structures, which achieve twice the voltage of Vin. Moreover, the PMOS drivers are inverter structures, accomplishing that PMOS can be closed strictly. The circuit structure is shown in Figure 7. 

The output power of the charge pump is proportional to frequency and capacitance, which can be expressed as

(8) $$P_{out}=f_{CLK}C_{D}V^{2}{}_{IN}$$

According to Formula (8), increasing the clock frequency and the charging node capacitance *C_D_* can improve output power. Nevertheless, at the same time, it is necessary to consider the dynamic loss caused by the parasitic capacitance charging and discharging, and the frequency or capacitance cannot be increased infinitely. The analysis in the previous section shows that the solution adopted in this paper is to pursue the maximum value of the output voltage to provide a stable voltage to the subsequent modules. The feedback MPPT control module tracks the maximum value of the voltage and gives the appropriate value of the clock frequency. Finally, we can maximize the efficiency of the entire system, and the output power of the PV cell may not be at the maximum power point at this time.

In the same way, the larger the flying capacitor, the greater the output power, but the sizeable flying capacitor causes the chip area to be too large, and when the capacitor is high in power, the larger capacitor has little effect on it, and finally through the simulation Optimize, we set the capacitance value to 200 pF. A four-phase time generation circuit, having a solid driving capability, needs to be designed due to large fly capacitors. The component parameters of the four-phase charge pump are illustrated in Table 1. 

**Figure 8:**
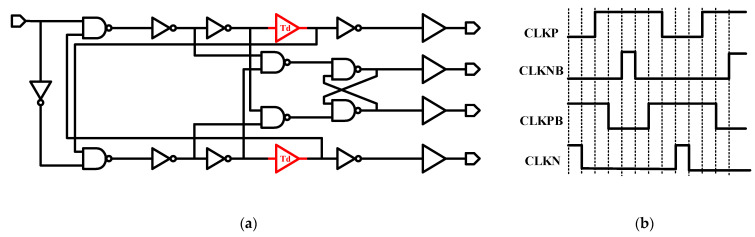
(**a**) Four-phase clock generation circuit; (**b**) Four-phase clock logic diagram.

Figure 8 shows the four-phase clock generation circuit. Two cross-coupling NAND are the basis of the module, and the delay block decides the dead-time of the four-phase clock. We optimize Td (the time of Delay dead-time) to decrease transmission consumption (by optimizing charge stagnation time).

### 3.2. Ultra-Low-Power P&O MPPT Algorithm and Control Block

**Figure 9:**
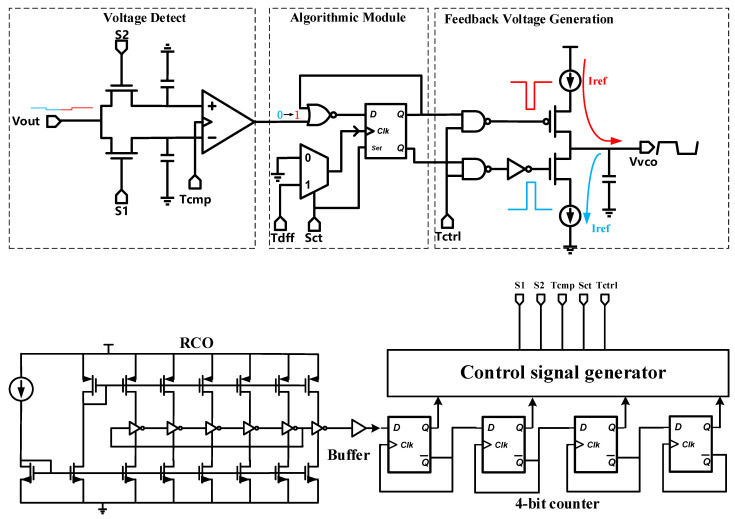
Schematic of MPPT control module and the control clock generation module.

As is depicted in Figure 9, the MPPT module is divided into three submodules: output voltage detection module, algorithmic module, and control voltage generation module. The whole module works in four steps. Firstly, the sampling process and hold process are established. *S1* and *S2* control *V_out_* in a period, and the current and previous time *V_out_* is stored in two input capacitors of the comparator, respectively. Secondly, the comparator in Figure 9, is a fast and dynamic comparator with ultra-low power consumption, is used to compare the voltage at the current time with the previous voltage to obtain the changing trend of the voltage. It can be observed that the changing trend of the output voltage and the direction of the voltage disturbance are in an XOR relationship from the logic block diagram of the P&O method. Then, the DFF can save the last disturbance direction, judge the next disturbance direction, perform the XOR operation to get the next disturbance direction, and then give the result to the subsequent logic gate. Finally, a structure based on the charge pump changes the voltage of the output node by charging and discharging the node capacitance to control the oscillation frequency of the VCO and then changes the system’s impedance. To minimize the power loss, we set up *I_ref_* = 10 nA, and the MPPT control accuracy is set to ∆*V_vco_* = 10 mV per cycle. The capacitance of MPPT can be known by Formula (9) and (10). The dissipation power of the MPPT module is lower than 1 uW.

(9) $$Q=I_{ref}\times \Delta t$$

(10) $$C=\frac{Q}{\Delta V_{vco}}=5pF$$

Figure 9 also shows the MPPT control clock generation module. The module provides the signal that needs to control the clock for the MPPT module. The initial clock of this design is generated by the ring oscillator (RCO), and the clock generates five kinds of control clocks through the operation of the four-bit counter (consists of DFF) and the logic control module. The current of the ring oscillator is provided by a nano-amp current reference circuit, the period of the clock signal generated by the oscillator is 5 μs, and the period of the output control signal is 80 μs.

### 3.3. Nano-Ampere Current Reference and Current Starvation VCO

The power management system (PMS) usually needs a current reference to provide a stable and independent power supply current [27,28]. Since the current drive capability of solar photovoltaic cells is very limited, the power loss of the internal module must be ultra-low. The nano-ampere current reference circuit proposed can solve the problem [29,30,31]. According to the contrary temperature characteristic of electrons and holes, the reference current has a weak temperature sensitivity. After the output voltage is established, the nano-ampere current reference circuit provides current to other modules. Besides, a relatively stable voltage can be provided by the reference circuit. The detector module gets the information of switching modes by comparing *V_ref_* and the control voltage of VCO. 

**Figure 10:**
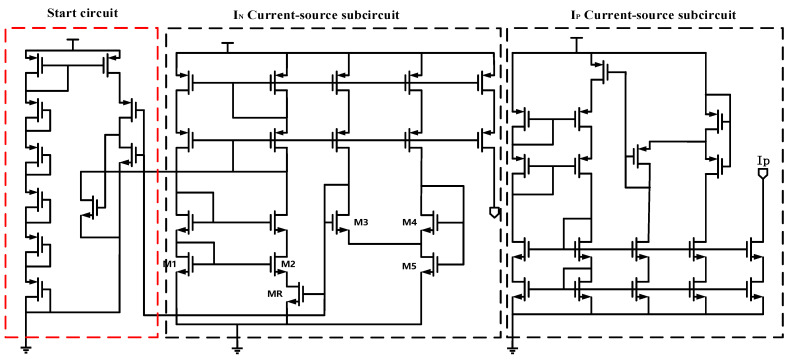
Schematic of Nano-ampere current reference.

Figure 10 shows the nano-ampere bias current reference circuits we designed. It includes a start circuit and Symmetrical *I_n_* and *I_p_* current-source circuit. To reduce the power consumption to the nano-ampere level, we all use MOS devices, and all MOS transistors work in the sub-threshold region (except MR working in a deep linear region).

When CMOS worked at subthreshold, current can be presented as

(11) $$I=I_{0}\exp(\frac{V_{GS}-V_{th}}{\zeta V_{T}})$$

where $V_{T}=\frac{KT}{q}$, ζ is a general factor and ζ>1.

When a nano-ampere current is generated, the resistance value needs to be very large, which occupies a large area. Thus, we select a NMOS that works in the linear region as the resistance, and the expression is

(12) $$R_{MR}=\frac{1}{\mu_{n}C_{OX}\frac{W}{L}(V_{GS}-V_{th})}$$

The proportion of MOS in this circuit is as follow

(13) $$M_{1}:M_{2}=k_{1}:k_{2};\;M_{3}:M_{4}=k_{3}:k_{4}$$

The drain-source voltage of MOS in the linear region is

(14) $$V_{ds,R}=\frac{nKT}{q}\ln(\frac{k_{2}}{k_{1}})$$

(15) $$V_{gs,R}=V_{gs,5}-V_{gs,4}+V_{gs,3}=V_{gs,5}+\frac{nKT}{q}\ln(\frac{k_{4}}{k_{3}})$$

where $V_{gs,R}=V_{th,5}$, Equation (15) can be rewritten as

(16) $$V_{gs,R}-V_{th,R}=V_{gs,5}+\frac{nKT}{q}\ln(\frac{k_{4}}{k_{3}})-V_{th,5}$$

The bias current is

(17) $$I_{bias}=\frac{V_{ds,R}}{R_{MR}}=\frac{\frac{nKT}{q}\ln(\frac{k_{2}}{k_{1}})}{1/(\mu_{n}C_{OX}{\left(\frac{W}{L}\right)}_{R}\frac{nKT}{q}\ln(\frac{k_{4}}{k_{3}})+V_{gs,5})}$$

Besides, PMOS and NMOS can be exchanged in the same way. That is, *I_p_* with holes as carriers can be designed. We adjust the size of the MOS. *I_n_* and *I_p_* are both 10nA, and the error is less than 150 pA.

**Figure 11:**
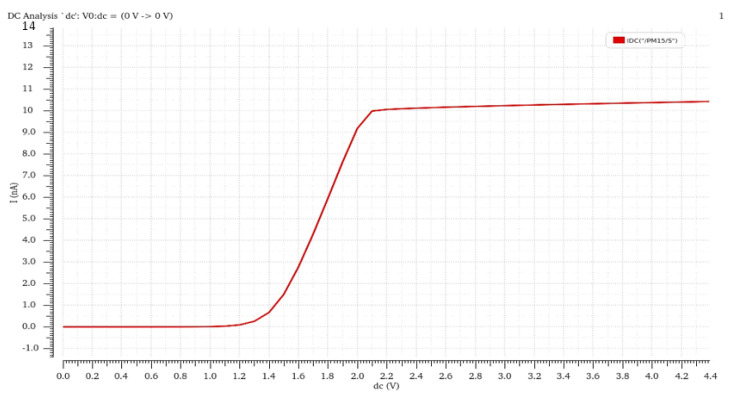
Relationship between the power supply voltage and output current.

The final current is related to the size of MR, the ratio of M3 and M4, and the ratio of M1 and M2. With the aid of SPICE, using the 0.35 um CMOS process, we simulated the relationship between the supply voltage. The output current is revealed in Figure 11. When Vin is more than 2 V, the bias current has a good PSRR.

**Figure 12:**
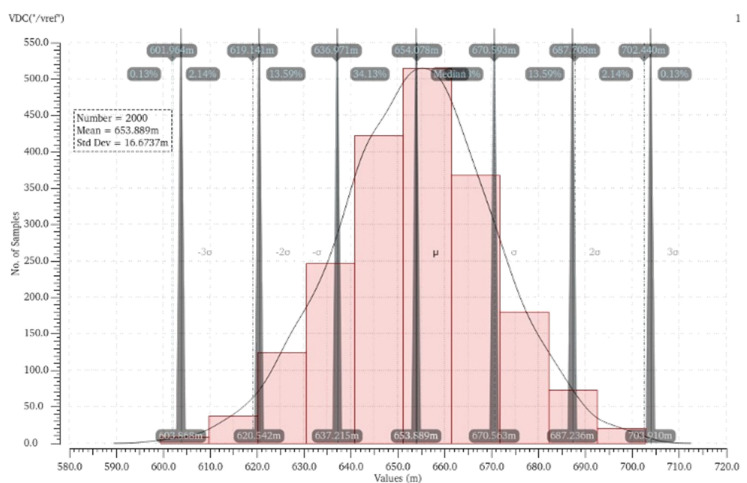
Distribution of output reference voltage with Monte Carlo simulations of 2000 runs.

A transistor and a source-couple pair can generate voltage reference. Figure 12 shows the Monte Carlo simulation of nano-ampere. The mean and deviation of *V_ref_* are 653.89 mV and 16.67 mV, respectively, and the coefficient of variation (σ/μ) is 2.549%.

**Figure 13:**
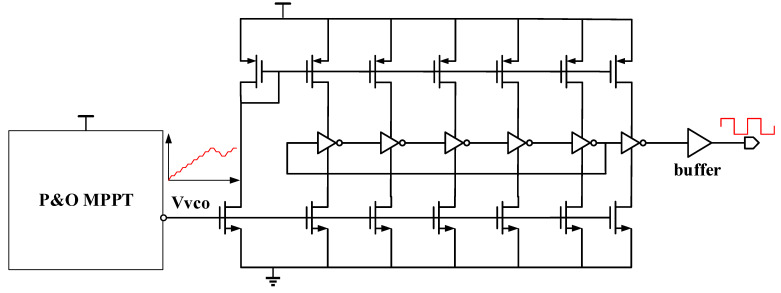
The circuit of the current-hungry voltage control oscillator.

This paper designs a current-hungry oscillator based on a ring oscillator. The structure can regulate the frequency of the charge pump quickly and consume low power. The current of each branch inverter can keep the same through a current mirror, thereby stabilizing the output and reducing power consumption. The structure is depicted in Figure 13. The delay time of the inverter is determined by the current and the size of the parasitic capacitance. Therefore, we can control the output frequency of the ring oscillator by controlling the current. 

The oscillator in this paper can ensure low power consumption and stable frequency, and frequency is expressed as

(18) $$f=\frac{1}{N}\times T_{D}$$

where *N* is the number of inverters and *T_D_* is a single inverter delay time.

**Figure 14:**
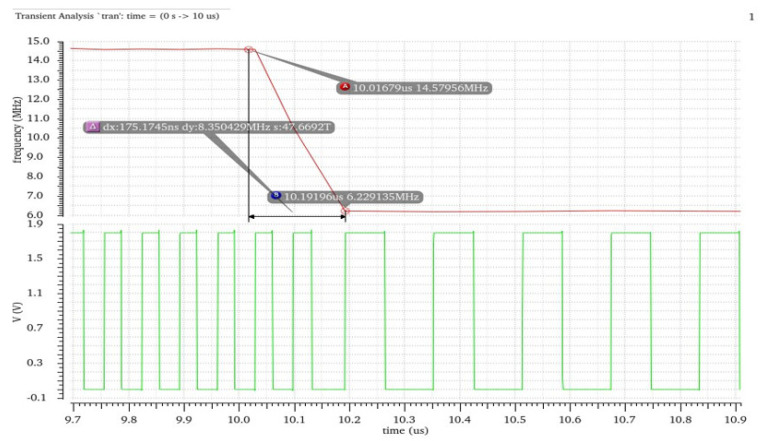
The simulation results of the current-hungry voltage control oscillator.

As Figure 14 plotted, a current-hungry VCO this paper proposed has a quick frequency response. When the control voltage is reduced from 450 mV to 400 mV, the frequency changes from 14.5 MHz to 6.22 MHz.

## 4. Experimental Results and Discussion

**Figure 15:**
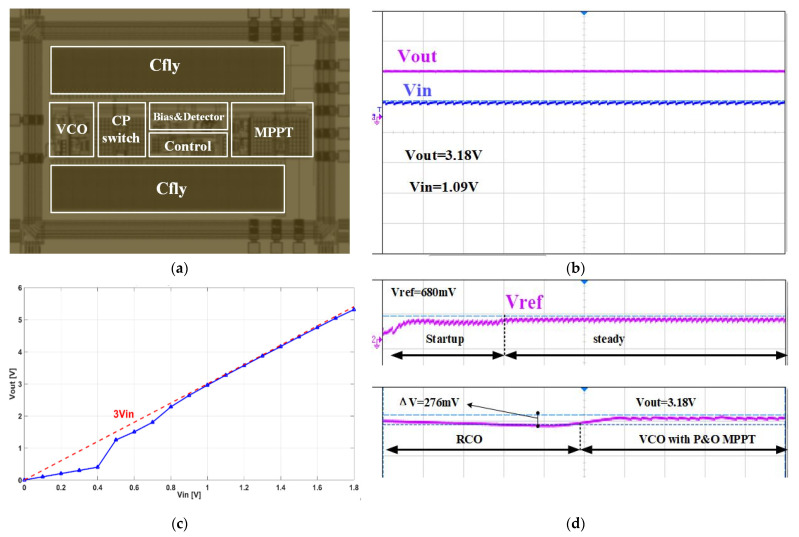
(**a**) The micro-photograph of VBC IC; (**b**) Measured waveform of output and input voltage; (**c**) Transfer characteristic of VBC; (**d**) Measured waveform of *V_ref_* and the output voltage in the switching process.

The VBC with MPPT IC this paper proposed was designed and fabricated in a 0.35 um CMOS-BCD process. The chip micrograph is shown in Figure 15a. The chip area is 3.15 mm × 2.43 mm. The interface IC includes a charge pump switch module, P&O MPPT control circuit module, feedback control module, nano-ampere current reference, and large area Cfly.

We set up A platform by linking the PV cell (mentioned in Section 1) with the chip to test its performance. Figure 15b depicts *V_in_* and *V_out_* in Oscilloscope when the sunlight density is 400 W/m^2^. The output voltage of the photovoltaic cell is 1.09 V, and the load resistance is 5 K. The output voltage can reach 3.18 V, and the ripple is less than 15 mV. 

When changing the sunlight intensity, VBC input voltage varied from 0 to 1.8 V. The transfer characteristic of VBC we measured is shown in Figure 15c by testing and collecting the output voltage data point. It can be observed that when Vin is more than 0.5 V, the output voltage is already very close to 3 Vin, and the calculated voltage conversion efficiency can reach 99.4%. The results show that the VBC can operate at a relatively low input voltage. Figure 15d shows the *V_ref_* and the mode switch process of VBC. When the output voltage is established, *V_ref_* stabilizes at 680 mV. Furthermore, the waveform depicts that P&O MPPT algorithm makes output voltage improve 276 mV.

**Figure 16:**
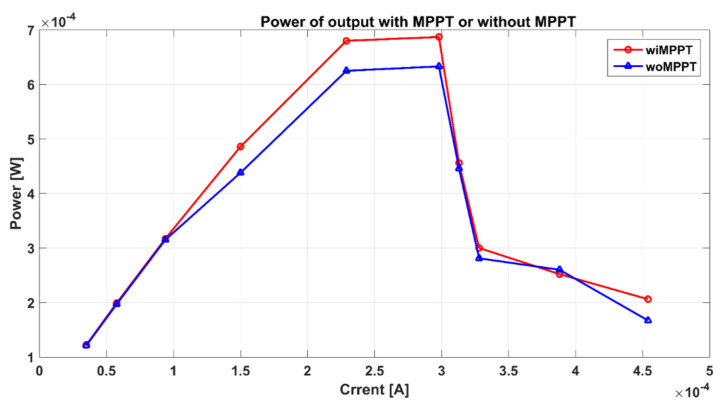
Power of output with MPPT and without MPPT.

MPPT results are measured by comparing the VBC power of output with MPPT and the one without MPPT, when the light intensity is 400 W/m^2^. The data points are shown as Figure 16. When the output current is less than 100 uA, the effect of MPPT is not significant because the structure based on RCO and CP has the function of tracking the max power point in linear mode. 

When the output current is ≈300 uA, P&O MPPT algorithm has the best effect, and we can know that output voltage has been improved by 8.53%.

**Figure 17:**
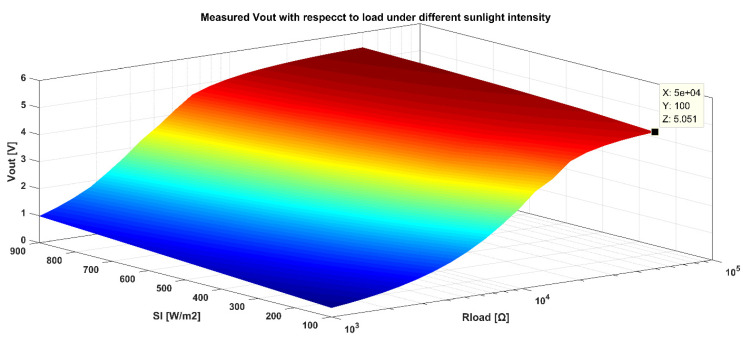
Measured output voltage System versus output resistance under different light intensity.

The performance of boosting voltage is depicted in Figure 17. Light intensity and output impedance decide the output voltage. The output voltage is measured and drawn in the relationship diagram (ignore the influence of temperature on the PV cell). It can be observed that the larger the load resistance, the larger the output voltage. When the load impedance is larger, the load current is smaller, and the light intensity has less influence on the output voltage. When SI is 100 W/m^2^, and the load impedance is 50 K, the output voltage of VBC is 5.051 V. Figure 17 also illustrates that an excessive output resistance can cause a failure to boost voltage.

**Figure 18:**
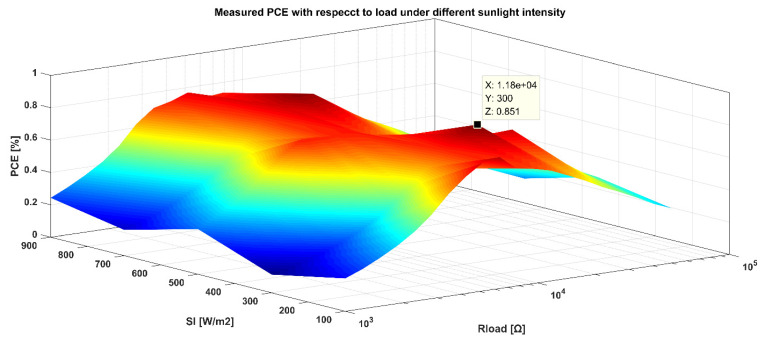
Measured output power under different light intensity.

Same as the output voltage, PV cells’ output and input power are measured under different light intensities and output resistance. The PCE is the output power divided by the input power of the PV cell, as shown in Figure 18. It can be seen from the figure that the light intensity varies from 100 W/m^2^ to 1000 W/m^2^. During the change, the higher the light intensity, the higher the power corresponding value of the highest conversion efficiency, the greater the turning point. The highest conversion efficiency is relatively large when the light intensity is higher than a particular value. When the light intensity is constant, the PCE will rise and decline as the output current increases. Taking the effective light intensity of 300 W/m^2^ as an example, it is about 85.1% when the output current is 297 μA. When the voltage is large, the current is small, and the efficiency is not high. Results are identical to the relationship among the PV characteristics of the photovoltaic cell, the loss of the charge pump, and the frequency.

The output power characteristic of the VBC is like the PV cell. Still, the corresponding peak impedance is not identical. The comparison result graph shows that the maximum output power point is not the same as the maximum transmission efficiency point. The reason is that if the output voltage (PV cell) continues to increase at the maximum output power point, the output power of the PV cell will drop faster than the output power of the system, which will result in a slight increase in PCE.

Table 2 summarizes performances of our design and others [7,8,12,13]. It can be concluded that the VBC we propose has a higher PCE than others, has a function of MPPT, and is more superior to other boost circuits in terms of the area. Thus, it is adapted to PV cells in WSNs. 

## 5. Discussion and Conclusions

It is known that in the field of wireless sensors, the Solar energy harvester is the most common renewable source. An efficient and tiny size power management system has a wide range of application scenarios. When the size of the PV cell is limited and the output voltage is small, a voltage boost is necessary. Besides, to minimize the size, a fully integrated design is urgently adopted. Compared to fossil works, the design we proposed meets the above application requirements, and many novel circuit structures are applied in this design to improve voltage convert performance.

In conclusion, this paper proposed a high PCE fully integrated voltage boost circuit with MPPT, with a higher input voltage range, and adapted to PV cells for WSNs. A 0.35 um CMOS BCD process is used to design this IC. The chip area is 3.15 mm × 2.43 mm. The whole system includes a novel four-phase high-efficiency charge pump module, MPPT control circuit module, feedback control module, nano-ampere current reference, etc. With transmission efficiency and load current as the goal, all modules are optimized, combined with simulation results. A PV cell model is also built, and MPPT algorithms to optimize power conversion efficiency are analyzed. Test results of IC show that when the light intensity is 400 W/m^2^, the output voltage of the photovoltaic cell is 1.09 V, the output voltage can reach 3.18 V, and the ripple is less than 15 mV. Calculation results show that the maximum voltage conversion efficiency is 99.4%. MPPT algorithm can help PCE to improve 8.53%. Regarding PCE, when the output current is 297 uA, the output efficiency can reach 85.1% at the maximum. 

## Figures and Tables

**Table 1:** The component parameters of the four-phase charge pump.

Device	Parameter
MN1/MN2	80/0.5
MP1/MP2	100/0.5
C1	5 pF
Cfly	200 pF

**Table 2:** Properties Summary and Comparison.

Properties	[7]	[8]	[12]	[13]	This Work
CMOS process	0.18 um	0.13 um	0.25 um	0.18 um	0.35 um
Chip area	1.6 × 1.1	0.066	3.4 × 3.4	1.6 × 1.7	3.15 × 2.43
Input voltage	0.5–0.6 V	0.15	0.5~2 V	<0.6 V	0.5–1.8 V
Output voltage	1.8/4.2 V	0.619	0~5 V	1.8/3 V	1.5–5.4 V
PCE	75.8%/49.1%	72.5%	87%	50%	85.1%
MPPT	yes	no	yes	yes	yes
Fully integrated	yes	yes	no	no	yes
Boost device	cap	cap	ind	-	cap

## Data Availability

Not applicable.

## References

[1] Pottie G.J., Clare L.P. (1999). Wireless Integrated Network Sensors: Towards Low Cost and Robust Self-Organizing Security Networks. Sensors, C3I, Information, and Training Technologies for Law Enforcement.

[2] Warneke B.A., Pister K.S.J. An ultra-low energy microcontroller for Smart Dust wireless sensor networks. Proceedings of the 2004 IEEE International Solid-State Circuits Conference (IEEE Cat. No.04CH37519).

[3] Doms I., Merken P., Mertens R., Hoof C.V. Integrated capacitive power-management circuit for thermal harvesters with output power 10 to 1000 µW. Proceedings of the 2009 IEEE International Solid-State Circuits Conference—Digest of Technical Papers.

[4] Wang W.S., Donnell T.O., Ribetto L., Flynn B.O., Hayes M., Mathuna C.O. Energy harvesting embedded wireless sensor system for building environment applications. Proceedings of the 2009 1st International Conference on Wireless Communication, Vehicular Technology, Information Theory and Aerospace & Electronic Systems Technology.

[5] Seeman M.D., Sanders S.R., Rabaey J.M. An Ultra-Low-Power Power Management IC for Wireless Sensor Nodes. Proceedings of the 2007 IEEE Custom Integrated Circuits Conference.

[6] Ozaki T., Hirose T., Nagai T., Tsubaki K., Kuroki N., Numa M. A 0.21-V minimum input, 73.6% maximum efficiency, fully integrated voltage boost converter with MPPT for low-voltage energy harvesters. Proceedings of the ESSCIRC 2014—40th European Solid State Circuits Conference (ESSCIRC).

[7] Ozaki T., Hirose T., Asano H., Kuroki N., Numa M.J.I. (2016). A fully-integrated, high-conversion-ratio and dual-output voltage boost converter with MPPT for low-voltage energy harvesting. IEEE J. Solid-State Circuits.

[8] Kim J., Mok P.K.T., Kim C. (2015). A 0.15 V Input Energy Harvesting Charge Pump with Dynamic Body Biasing and Adaptive Dead-Time for Efficiency Improvement. IEEE J. Solid-State Circuits.

[9] Yu G., Chew K.W.R., Sun Z.C., Tang H., Siek L. (2015). A 400 nW Single-Inductor Dual-Input–Tri-Output DC–DC Buck–Boost Converter with Maximum Power Point Tracking for Indoor Photovoltaic Energy Harvesting. IEEE J. Solid-State Circuits.

[10] Uprety S., Lee H. 22.5 A 93%-power-efficiency photovoltaic energy harvester with irradiance-aware auto-reconfigurable MPPT scheme achieving >95% MPPT efficiency across 650 µW to 1 W and 2.9 ms FOCV MPPT transient time. Proceedings of the 2017 IEEE International Solid-State Circuits Conference (ISSCC).

[11] Huang P., Kuo T. (2020). A Reconfigurable and Extendable Single-Inductor Single-Path Three-Switch Converter for Indoor Photovoltaic Energy Harvesting. IEEE J. Solid-State Circuits.

[12] Qiu Y., Liempd C.V., Veld B.O.h., Blanken P.G., Hoof C.V. 5 μW-to-10 mW input power range inductive boost converter for indoor photovoltaic energy harvesting with integrated maximum power point tracking algorithm. Proceedings of the 2011 IEEE International Solid-State Circuits Conference.

[13] Wu X., Shi Y., Jeloka S., Yang K., Lee I., Lee Y., Sylvester D., Blaauw D. (2017). A 20-pW Discontinuous Switched-Capacitor Energy Harvester for Smart Sensor Applications. IEEE J. Solid-State Circuits.

[14] Brito M.A.G.d., Galotto L., Sampaio L.P., e Melo G.D.A., Canesin C.A. (2013). Evaluation of the Main MPPT Techniques for Photovoltaic Applications. IEEE Trans. Ind. Electron..

[15] Sher H.A., Murtaza A.F., Noman A., Addoweesh K.E., Al-Haddad K., Chiaberge M. (2015). A New Sensorless Hybrid MPPT Algorithm Based on Fractional Short-Circuit Current Measurement and P&O MPPT. IEEE Trans. Sustain. Energy.

[16] Li S. (2019). A variable-weather-parameter MPPT control strategy based on MPPT constraint conditions of PV system with inverter. Energy Convers. Manag..

[17] Liu H.-D., Lin C.-H., Pai K.-J., Lin Y.-L. (2018). A novel photovoltaic system control strategies for improving hill climbing algorithm efficiencies in consideration of radian and load effect. Energy Convers. Manag..

[18] Hui S., Tsui C.Y., Ki W.H. (2009). The Design of a Micro Power Management System for Applications Using Photovoltaic Cells with the Maximum Output Power Control. IEEE Trans. Very Large Scale Integr. (VLSI) Syst..

[19] Kabalci E., Boyar A. Design and Comparison of MPPT Controllers with Fuzzy Logic and Particle Swarm Optimization for PV Power Conversion. Proceedings of the 2020 2nd International Conference on Control Systems, Mathematical Modeling, Automation and Energy Efficiency (SUMMA).

[20] Chen P., Wu C., Lin K. 20.10 A 50 nW-to-10 mW output power tri-mode digital buck converter with self-tracking zero current detection for photovoltaic energy harvesting. Proceedings of the 2015 IEEE International Solid-State Circuits Conference—(ISSCC) Digest of Technical Papers.

[21] Priyadarshi N., Padmanaban S., Maroti P.K., Sharma A. (2019). An Extensive Practical Investigation of FPSO-Based MPPT for Grid Integrated PV System under Variable Operating Conditions with Anti-Islanding Protection. IEEE Syst. J..

[22] Liu X., Ravichandran K., Sánchez-Sinencio E. (2018). A Switched Capacitor Energy Harvester Based on a Single-Cycle Criterion for MPPT to Eliminate Storage Capacitor. IEEE Trans. Circuits Syst. I Regul. Pap..

[23] Jung J.H., Hong S.K., Kwon O.K. (2020). A Highly Reliable SIMO Converter Using Hybrid Starter and Overcharging Protector for Energy Harvesting Systems. IEEE Access.

[24] Talkhooncheh A.H., Yu Y., Agarwal A., Kuo W., Emami A. A Fully-Integrated Biofuel-Cell-Based Energy Harvester with 86% Peak Efficiency and 0.25 V Minimum Input Voltage Using Source-Adaptive MPPT. Proceedings of the 2020 IEEE Custom Integrated Circuits Conference (CICC).

[25] Aloulou R., Lucas De Peslouan P.O., Mnif H., Alicalapa F., Lan Sun Luk J.D., Loulou M. (2016). A power management system for energy harvesting and wireless sensor networks application based on a novel charge pump circuit. Int. J. Electron..

[26] Favrat P., Deval P., Declercq M.J. (1998). A high-efficiency CMOS voltage doubler. IEEE J. Solid-State Circuits.

[27] Osaki Y., Hirose T., Kuroki N., Numa M. (2011). Temperature-Compensated NanoAmpere Current Reference Circuit with Subthreshold MetalOxideSemiconductor Field-Effect Transistor Resistor Ladder. Jpn. J. Appl. Phys..

[28] Duan Q., Wang X., Huang S., Ding Y., Meng Z., Shi K.J. (2019). 0.55–1.8 V, 7.5 nW, 225.5 mV, CMOS-only subthreshold voltage reference. Electron. Lett..

[29] Osaki Y., Hirose T., Kuroki N., Numa M. Nano-ampere CMOS current reference with little temperature dependence using small offset voltage. Proceedings of the IEEE International Midwest Symposium on Circuits & Systems.

[30] Ueno K., Hirose T., Asai T., Amemiya Y. (2009). A 300 nW, 15 ppm/°C, 20 ppm/V CMOS Voltage Reference Circuit Consisting of Subthreshold MOSFETs. IEEE J. Solid-State Circuits.

[31] Lacorte W.B., Manalo R.M. A Sub-1-V, High PSRR, Subthreshold MOSFET-only Voltage Reference for Wireless Sensor Networks. Proceedings of the TENCON 2019—2019 IEEE Region 10 Conference (TENCON).

